# Evaluation of the Importance of VlsE Antigenic Variation for the Enzootic Cycle of *Borrelia burgdorferi*

**DOI:** 10.1371/journal.pone.0124268

**Published:** 2015-04-20

**Authors:** Artem S. Rogovskyy, Timothy Casselli, Yvonne Tourand, Cami R. Jones, Jeb P. Owen, Kathleen L. Mason, Glen A. Scoles, Troy Bankhead

**Affiliations:** 1 Department of Veterinary Microbiology and Pathology, Washington State University, Pullman, Washington, United States of America; 2 Department of Entomology, Washington State University, Pullman, Washington, United States of America; 3 Animal Disease Research Unit, USDA-ARS, Washington State University, Pullman, Washington, United States of America; 4 Paul G. Allen School for Global Animal Health, Washington State University, Pullman, Washington, United States of America; University of Kentucky College of Medicine, UNITED STATES

## Abstract

Efficient acquisition and transmission of *Borrelia burgdorferi* by the tick vector, and the ability to persistently infect both vector and host, are important elements for the life cycle of the Lyme disease pathogen. Previous work has provided strong evidence implicating the significance of the *vls* locus for *B*. *burgdorferi* persistence. However, studies involving *vls* mutant clones have thus far only utilized *in vitro*-grown or host-adapted spirochetes and laboratory strains of mice. Additionally, the effects of *vls* mutation on tick acquisition and transmission has not yet been tested. Thus, the importance of VlsE antigenic variation for persistent infection of the natural reservoir host, and for the *B*. *burgdorferi* enzootic life cycle in general, has not been examined to date. In the current work, *Ixodes scapularis* and *Peromyscus maniculatus* were infected with different *vls* mutant clones to study the importance of the *vls* locus for the enzootic cycle of the Lyme disease pathogen. The findings highlight the significance of the *vls* system for long-term infection of the natural reservoir host, and show that VlsE antigenic variability is advantageous for efficient tick acquisition of *B*. *burgdorferi* from the mammalian reservoir. The data also indicate that the adaptation state of infecting spirochetes influences *B*. *burgdorferi* avoidance from host antibodies, which may be in part due to its respective VlsE expression levels. Overall, the current findings provide the most direct evidence on the importance of VlsE for the enzootic cycle of Lyme disease spirochetes, and underscore the significance of VlsE antigenic variation for maintaining *B*. *burgdorferi* in nature.

## Introduction

*Borrelia burgdorferi* is the causative bacterial agent of Lyme disease, which can clinically present as arthritis, carditis, and/or neurological disorders [1]. In nature, *B*. *burgdorferi* is maintained in an enzootic life cycle that involves an arthropod vector and small vertebrate host [1–4]. In North America, *B*. *burgdorferi* is transmitted mainly by the tick vectors, *Ixodes scapularis* and *Ixodes pacificus*. *Peromyscus leucopus* mice are considered the primary vertebrate reservoir, and *Peromyscus maniculatis* has also been shown to be a competent host in nature [1,5–9]. *Ixodes* larvae acquire spirochetes when feeding on an infected host, and *B*. *burgdorferi* is subsequently transmitted when infected nymphs feed on young uninfected animals [10]. Transmission from infected nymphs of one cohort to larvae of another through reservoir hosts is believed to be largely responsible for maintenance of *B*. *burgdorferi* in nature [11].

Efficient *B*. *burgdorferi* acquisition and transmission by the tick vector, and the ability to persistently infect both vector and host, are important elements for the life cycle of the Lyme pathogen [1,12]. Previous studies involving laboratory strains of mice have provided strong evidence implicating the significance of the *vls* locus for *B*. *burgdorferi* persistence [13–15]. The locus consists of the *vlsE* expression site and a tandem array of 15 silent cassettes, all of which are located near the right telomere end of the linear plasmid, lp28-1 [16–18]. Gene conversion events at the *vls* locus result in sequence variation of the 35kDa surface lipoprotein, VlsE [16,17]. Changes in the DNA sequence of *vlsE* have been shown to occur primarily within the central variable region of the expression site. Genetic variations in *vlsE* have been detected as early as four days after infection of mice [19], and have been observed to continue throughout infection [20]. Previous studies have also found that antibodies specific for the variable regions of VlsE are produced during experimental infection of mice [21]. An interesting feature of *vlsE* antigenic switching is that it appears to only occur during mammalian infection [16,19], which may suggest that some host factor(s) are required to activate the *vls* recombination process.

Studies involving the *vls*-resident plasmid, lp28-1, were the first to provide evidence for the role of the *vls* system in immune avoidance [22,23]. Clones lacking lp28-1 exhibit the ability to disseminate to distal tissue sites, but are unable to persist during infection of the murine host. However, lp28-1-deficient spirochetes are capable of long-term survival in severe-combined immunodeficient (SCID) mice that lack an effective antibody response [24,25]. It has also been shown that dialysis membrane chambers that restrict host antibody access to spirochetes allow lp28-1-deficient isolates to persist in the peritoneal cavity of rats [25]. Complementation of an lp28-1-deficient clone with only the *vlsE* gene (in the absence of any *vls* silent cassettes) does not enable spirochetes to establish persistent infection in an immunocompetent murine host [14]. Direct evidence for the role of VlsE antigenic variation in persistence was provided by the generation of a genetic deletion of the *vls* locus [13,26]. This *vls*-deficient clone (Δ*vlsE*) was shown to be completely cleared in immunocompetent C3H mice by day 21 post infection. Finally, the variable VlsE-generating capacity of the *vls* locus has recently been shown to be essential for host reinfection [15]. The results from that study demonstrated that variable VlsE is required for host-adapted *B*. *burgdorferi* to reinfect C3H mice that have previously cleared infection with the *vls* knockout strain. Moreover, the presence of an intact *vls* locus is required for spirochetes to escape Δ*vlsE*-specific antibodies that otherwise are able to prevent infection by this mutant *B*. *burgdorferi* clone.

With respect to tick acquisition and transmission, *I*. *scapularis* larvae or nymphs artificially infected with *B*. *burgdorferi* clones lacking lp28-1 have been shown to be successfully infected at levels similar to wild-type *B*. *burgdorferi*, suggesting that the *vls* locus is not necessary for efficient tick colonization [27,28]. In addition, these same lp28-1 minus spirochetes can be transmitted to naïve mice by infected nymphs. Despite this evidence for the lack of any role for the *vlsE*-resident plasmid in tick acquisition and transmission, studies involving the use of a *vls* knockout mutant to assess the effects of *vls* mutation on these processes have not been examined to date [15]. Additionally, mouse studies involving *vls* mutant *B*. *burgdorferi* clones have thus far only utilized *in vitro*-grown or host-adapted spirochetes for infection of laboratory strains of mice. Results from *Mus musculus* are often used to justify clinical trials, but are considered to be insufficiently predictive to answer ecology-related questions of Lyme *Borrelia* [29,30].

In the present work, *Ixodes scapularis* and *Peromyscus maniculatus* were utilized as a model to study the significance of VlsE variation for the *B*. *burgdorferi* enzootic cycle. Specifically, this work examines the importance of VlsE antigenic variation for *B*. *burgdorferi* to establish infection in both natural murine and arthropod hosts by taking advantage of previously generated *B*. *burgdorferi vls* mutants. The results show that a mutant clone expressing a non-variable form of VlsE exhibits a reduced rate of tick acquisition from both infected C3H and *Peromyscus* mice compared to the wild-type and VlsE-deficient *B*. *burgdorferi* clones. Impaired rates are not observed when uninfected tick larvae have been fed on infected SCID mice, suggesting that host antibodies may be responsible for the reduced acquisition of *vlsE* mutant spirochetes. Tick transmission of *B*. *burgdorferi* to naïve mice, however, does not differ between the wild-type and *vlsE* mutant *B*. *burgdorferi* clones. The results also demonstrate that the presence of the intact *vls* locus is required to ensure persistence during infection of the competent reservoir host, which is consistent with previous findings involving laboratory mouse models [13]. Overall, the study provides the first direct evidence of the importance of the *vls* locus during the enzootic cycle of Lyme spirochetes, and highlights the relevance of VlsE antigenic variation for maintaining *B*. *burgdorferi* in nature.

## Materials and Methods

### Ethics statement

The experimental procedures involving *Mus musculus* strains of inbred mice and *Peromyscus maniculatus* mice were carried out in accordance with the American Association for Accreditation of Laboratory Animal Care (AAALAC) protocol and the institutional guidelines set by the Office of Campus Veterinarian at Washington State University (Animal Welfare Assurance A3485-01 and USDA registration number 91-R-002). Washington State University AAALAC and institutional guidelines are in compliance with the U.S. Public Health Service Policy on Humane Care and Use of Laboratory Animals. *Mus musculus* inbred mice were maintained at Washington State University (Pullman, WA, USA) in an AAALAC-accredited animal facility. The Washington State University Institutional Animal Care and Use Committee reviewed and approved the animal protocols associated with the current studies. The experiments that involved *Peromyscus maniculatus* mice were conducted at the University of Idaho (Moscow, ID, USA) using standard protocols approved by the University of Idaho Institutional Animal Care and Use Committee.

### Bacterial strains and culture conditions

*B*. *burgdorferi* strain B31-A3 [31] was kindly provided by Patti Rosa. The B31-A3Δ*vls* and B31-A3 lp28-1Δ*vls*::*vlsE* clones were previously generated and characterized in laboratory strains of mice [13,15,32]. The infectious *B*. *burgdorferi* 297 strain [33] was a kind gift from Scott Samuels by way of Michael Norgard. All *B*. *burgdorferi* clones were cultivated in liquid Barbour—Stoenner—Kelly II medium (BSK) supplemented with 6% rabbit serum (Cedarlane Laboratories, Burlington, NC) and incubated at 35°C under 2.5% CO_2_. Plasmid content for each clone was determined by PCR using plasmid-specific primers as previously described [23].

### Murine models and challenge with *B*. *burgdorferi*

*Peromyscus maniculatus bairdii* (*P*. *maniculatus*) mice were obtained from the *Peromyscus* genetic stock center at University of South Carolina (Columbia, SC, USA) and maintained in the United States Department of Agriculture facility at the University of Idaho (Moscow, ID, USA). *P*. *maniculatus* mice of 8–12 weeks of age were used in the experiments. When *in vitro*-grown *B*. *burgdorferi* clones were used, each *P*. *maniculatus* mouse was subcutaneously inoculated with 1.1x10^5^ total spirochetes (in our hands, this inoculum dosage was found to guarantee 100% infectivity in these mice).

Male C.B-17/IcrHsd-*Prkdc* ^*scid*^ (SCID) and C3H/HeNHsd (C3H) of 4–6 weeks of age were purchased from Harlan Laboratories (Indianapolis, IN, USA). SCID and C3H mice were subcutaneously infected with *in vitro*-grown *B*. *burgdorferi* at 1.1x10^4^ spirochetes per mouse. *B*. *burgdorferi* clones from frozen glycerol stock were passaged no more than two times *in vitro* prior to use for mouse infection. The infectivity of each *in vitro*-derived *B*. *burgdorferi* clone utilized for secondary challenge was tested on naïve mice (see Results for details).

In order to obtain host-adapted *B*. *burgdorferi* clones, SCID mice were needle inoculated with *B*. *burgdorferi*. After verification of infection, ear tissues were harvested from infected SCID mice at day 28 post infection as previously described [15,32,34] and stored at -80°C until use. To challenge mice with host-adapted *B*. *burgdorferi*, ear pinnae were excised into small, circular pieces (3 mm in diameter) by a sterile ear punch and subcutaneously inserted via a skin incision in the lumbar region (two pieces per mouse).

Infection was confirmed by culturing approximately 50 ul of blood aseptically sampled from a mouse via maxillary bleed in 3 ml of BSK containing *Borrelia* antibiotic cocktail (0.02 mg ml^-1^ phosphomycin, 0.05 mg ml^-1^ rifampicin and 2.5 mg ml^-1^ amphotericin B). In order to monitor the progress of infection, ear, heart, bladder, and tibiotarsal joint tissues were aseptically harvested at various time points post infection and cultured in BSK supplemented with the antibiotic cocktail. Polystyrene tubes (8 ml; Becton Dickinson Labware, USA) were used for culturing blood (50 ul of blood in 3 ml of BSK medium) and cardiac tissue (approximately 1/2 heart in 3.0 ml of BSK medium), and 2.0 ml polypropylene microcentrifuge tubes (Fisherbrand, USA) were utilized for the other tissues (approximately 1/2 bladder, a tibiotarsal joint, or ear tissue (approximately 3 mm in diameter) in 1.0 ml of BSK medium. BSK media with mouse tissues were incubated at 35°C under 2.5% CO_2_. The presence of viable spirochetes was verified by dark-field microscopy.

### Quantification of bacterial burden in murine blood

Seven days post-infection, mice were anesthetized by isoflurane inhalation, and 200 uL of blood was collected from each of the saphenous and submandibular veins. Blood from each site was diluted in liquid BSK (8 ml total per animal) containing Borrelia antibiotic cocktail. Cultures were incubated at 35°C for approximately 12 hours to allow for settling of blood cells. Culture was separated from blood cells by aspiration, and plated by limiting dilution on semi-solid BSK containing 1% agarose. Plates were incubated for 7 days at 35°C, at which time colony forming units were enumerated. A subset of isolated colonies from each strain were harvested by sterile pipet tip and cultured in liquid BSK for PCR plasmid profile screening as previously described [23].

### Ticks

Uninfected *Ixodes scapularis* larvae were derived from a pathogen-free tick colony maintained at Oklahoma State University (Stillwater, OK, USA). To generate *B*. *burgdorferi*-infected *I*. *scapularis* nymphs, naïve C3H mice were subcutaneously inoculated with *in vitro*-grown wild-type or *vls* mutant clones at 1.1x10^4^ total spirochetes per animal. At day 6 post infection, larval ticks were fed upon the ketamine-anesthetized animals. Ketamine cocktail was prepared by combining xylazine (Lloyd, Shenandoah, IA, USA) and ketamine (Phoenix Pharmaceutical, St. Joseph, MO, USA) with final concentrations being 1.95 and 15.6 mg/ml, respectively and diluting 1:4 with PBS. Mice were intramuscularly injected with a dose of 100 ul of cocktail per 20 g of body weight. Mouse infection was verified by culturing blood sampled from each mouse at day 6 post infection prior to tick application.

Approximately 200 larval ticks were placed on each mouse. After 48 hours, each animal was transferred to a wired-bottomed cage placed over a tray filled with distilled water. Replete larvae were collected from the water every 12 hours for a total of 72 hours. Larvae were washed with running water, blotted dry and stored in screened vials at 25°C and 99% humidity until larvae molted into nymphs (4–6 weeks). Ten to twenty randomly chosen unfed nymphs infected with each *B*. *burgdorferi* clone were frozen at -20°C for quantitative-PCR analysis. In order to determine the infectivity rate of infected ticks, replete larvae or unfed nymphs were individually crushed and cultured in BSK media supplemented with the antibiotic cocktail (see above). Tick tissues were incubated at 35°C under 2.5% CO_2_. The presence of viable spirochetes was verified by dark-field microscopy. Tick-transmitted infection of mice was carried out by applying 5–8 unfed, infected nymphs onto naïve mice and allowing ticks to feed to repletion. The above feeding experiments involving both larval and nymphal ticks were repeated a second time in order to account for any biological variation.

### Immunoblotting

*B*. *burgdorferi* B31-A3 was cultured in BSK media to late stationary phase. Spirochetes were counted, pelleted by centrifugation at 6,000xg for 10 min at 4°C, and then washed twice with ice-cold PBS. After removal of PBS, the cells were suspended in sodium dodecyl sulfate (SDS)-polyacrylamide gel electrophoresis sample buffer (100 mM Tris [pH 6.8], 2% SDS, 5% β-mercaptoethanol, 10% glycerol, 0.01% bromophenol blue), and incubated at 95°C for 10 min. Approximately 1x10^6^ cells were loaded into each sample lane of a 15% acrylamide minigel. Resolved proteins were transferred onto polyvinylidene fluoride membrane with a pore size of 0.45 um (Immobilon-P, Millipore, Billerica, MA, USA). The blot was blocked with 5% nonfat dry milk in PBS for 18 hours at 4°C and then incubated in the same solution supplemented with 1:1,000 diluted sera obtained from culture-negative or -positive (positive control) *P*. *maniculatus* blood at day 28 post infection or preimmune sera (negative control) for 1 hour. After 4 washes of 10 min each with TBST, anti-*Borrelia* antibodies were detected using anti-mouse HorseRadish Peroxidase (HRP)-conjugated secondary antibody (Jackson ImmunoResearch Laboratories, West Grove, PA, USA) diluted to 1:1,000 in TBST for 30 min. The blot was washed 3 times in TBST for 10 min each, followed by a last wash in nano-pure water. The blots were visualized by Enhanced ChemiLuminescence (ECL) development.

### Passive immunization of mice

In order to generate immune sera, *P*. *maniculatus* mice were subcutaneously inoculated with *B*. *burgdorferi* B31-A3 lp28-1Δ*vls* at 1.1x10^5^ total spirochetes per mouse. Blood from infected mice was collected via cardiac puncture at day 28 post infection. Collected blood was kept at room temperature for 60 min and then centrifuged at 6,000xg for 15 min to remove sera from the blood cell pellet. Immune sera from B31-A3 lp28-1Δ*vls*-infected mice were pooled from a total of six mice, and pooled preimmune sera were derived from three age-matched, naïve *P*. *maniculatus* mice. All sera were stored at -20°C until required for passive immunization. At the time of experiment, immune or preimmune sera were diluted to 1:3 with sterile saline and filter-sterilized by passage through 0.22 um syringe filter. SCID mice were then treated with 150 ul of the diluted immune sera via subcutaneous and intraperitoneal needle inoculations at each site.

### qPCR analysis

Unfed nymphs infected with each *B*. *burgdorferi* clone were individually snap-frozen in liquid nitrogen and ground in a 1.5 ml polypropylene microcentrifuge tube (Fisherbrand, USA) with a polypropylene pestle (Bel-Art Products, Wayne, NJ, USA). Tick DNA was extracted using the DNeasy Minikit according to the manufacturer’s protocol (Qiagen, Germantown, MD, USA) and stored at -20°C. The previously constructed pJET2.1::*flaB* [35] was used to generate absolute standards using primers and internal probe for *flaB* as described previously [36]. DNA concentrations determined by measuring the optical density at 260 nm were converted to the respective copy numbers.

The CFX96 Touch Real-Time PCR detection system (Bio-Rad Laboratories, Hercules, CA, USA) was utilized to perform qPCR analysis. qPCR was carried out in 20ul reaction mixtures containing 1XSsoFast Probes Supermix (Bio-Rad Laboratories, Hercules, CA, USA) as previously described [35]. Specifically, DNA standards containing 10^4^ to 10^0^ copies per well of the *flaB* gene were run on each plate. Both standards and samples were amplified in triplicate. The amplification program consisted of (i) heating at 95°C for 2 min for polymerase activation and DNA denaturation, (ii) amplification for 40 cycles with denaturation at 95°C for 10 s and extension and annealing at 60°C. Plate reading was performed at 60°C. The average DNA copy numbers of *flaB* for each tick DNA sample were calculated from triplicate wells.

### Statistical analysis

A one-tailed Fisher’s exact test and two tailed t-test were used for comparison of mouse and tick groups, respectively. A *p* value of <0.05 was considered significantly different.

## Results

### The *vls* locus is essential for persistent infection of a natural reservoir host

To examine the importance of the *vls* locus for the *B*. *burgdorferi* enzootic cycle, two previously generated *vls B*. *burgdorferi* mutant clones, B31-A3 lp28-1Δ*vls* (Δ*vlsE*; containing a deletion of the entire *vls* locus) and B31-A3 lp28-1Δ*vls*::*vlsE* (s*vlsE*; expresses a static, non-variable version of VlsE) were used to infect the competent mammalian *B*. *burgdorferi* reservoir and arthropod vector, *P*. *maniculatus* and *I*. *scapularis*, respectively. Prior studies using inbred laboratory strains of mice have demonstrated that infection of immunocompentent, but not immunodeficient, mice by either mutant clone is cleared by day 21 post infection [13,15]. The non-variable s*vlsE* mutant possesses only the *vlsE* gene with its native promoter (lacking the silent cassette region) on the lp28-1 plasmid, and the VlsE protein is expressed on the spirochete surface *in vitro*, albeit at lower levels than the wild type [15]. Plasmid profile analysis of the parental B31-A3 wild-type (wtB31) and *vls* mutant clones was carried out via PCR prior to mouse infection experiments to ensure retention of *B*. *burgdorferi* plasmids, including those essential for infectivity [23]. All clones contained the full plasmid profile, with the exception of cp9. The cp9 plasmid is normally absent from the parental wtB31 clone, and is not necessary for infection or pathogenesis [31].

To characterize the *B*. *burgdorferi* clones in *P*. *maniculatus*, naive mice (3 or 5 animals per group) were subcutaneously infected with either wild-type (wtB31 or 297) or *vls* mutant (Δ*vlsE* or s*vlsE*) *B*. *burgdorferi* (see Table 1). The clinical isolate 297 is a phylogenetically distinct strain of *B*. *burgdorferi* compared to the B31 type strain as determined using multiple genetic markers [33,37]. All mice were needle inoculated with a total of 1.1x10^5^ spirochetes per mouse. Blood samples from day 7 post infection produced positive cultures for spirochetes from all mice infected with either wild-type or *vls* mutant clones (Table 2). However, tissue samples (ear, heart, bladder and joint) from day 21 and beyond were positive for spirochetes in only those mice that had been infected with wild-type *B*. *burgdorferi*, suggesting that all *P*. *maniculatis* mice inoculated with either *vls* mutant clone had successfully cleared infection.

**Table 1:** *B*. *burgdorferi* clones used in the study.

*B*. *burgdorferi* clone	*vls2-16* ^a^	*vlsE*	Reference
B31-A3 (wtB31)	+	+	[31]
B31-A3 lp28-1Δ*vls* (Δ*vlsE*)	-	-	[13]
B31-A3 lp28-1Δ*vls*::*vlsE* (s*vlsE*)	-	+	[15]
297	ND^b^	+	[33]

^a^ *vls2-16* denotes silent cassettes of the *vls* locus.

^b^ silent cassette region in this strain has not been determined to date.

**Table 2:** Infectivity of in vitro-grown *B*. *burgdorferi* clones in *P*. *maniculatus* mice.

Tissue collected (at day post infection)	Naïve *P*. *maniculatus* mice infected with *in vitro*-grown clones:			
	wtB31	297	Δ*vlsE*	s*vlsE*
Blood (day 7)	3/3^a^	3/3	5/5	5/5
Ear (day 21)	3/3	3/3	0/5	0/5
Ear (day 28)	3/3	3/3	0/5	0/5
Heart (day 28)	3/3	3/3	0/5	0/5
Bladder (day 28)	3/3	3/3	0/5	0/5
Joint (day 28)	3/3	3/3	0/5	0/5

^a^ Values listed correspond to numbers of cultures positive/numbers tested.

In order to assess whether reservoir mice could clear *Ixodes* tick-transmitted infection by *vls* mutant *B*. *burgdorferi*, *I*. *scapularis* nymphs infected with either wtB31, 297, Δ*vlsE* or s*vlsE* (5–8 ticks per mouse) were applied onto naive *P*. *maniculatus* mice (5 animals per group). Spirochetemia was detected by culture at day 7 post infection in all animals that were fed on by ticks infected with either wild-type or mutant *B*. *burgdorferi*, indicating dispensability of the *vls* system for tick-mediated *B*. *burgdorferi* transmission into *P*. *maniculatis* mice (Table 3). Again, as opposed to either wild-type strain (B31 or 297), both *vls* mutant clones were cleared by day 21 post infection in all mice tested, demonstrating that the *vls* locus is required for *Ixodes* tick-transmitted *B*. *burgdorferi* to establish a persistent infection in the natural host reservoir. Together, the above findings are in full agreement with previously published data obtained from laboratory strains of *Mus musculus* [13–15,22,23], and highlight the importance and relevance of VlsE antigenic variability for long-term infection in the natural reservoir host.

**Table 3:** Infectivity of nymph-transmitted *B*. *burgdorferi* clones in *P*. *maniculatus* mice.

Tissue collected (at day post infection)	Naïve *P. maniculatus* mice infected with nymph-transmitted clones			
	wtB31	297	Δ*vlsE*	s*vlsE*
Blood (day 7)	5/5^a^	5/5	5/5	5/5
Ear (day 21)	5/5	5/5	0/5	0/5
Ear (day 28)	5/5	5/5	0/5	0/5
Heart (day 28)	5/5	5/5	0/5	0/5
Bladder (day 28)	5/5	5/5	0/5	0/5
Joint (day 28)	5/5	5/5	0/5	0/5

^a^ Values listed correspond to numbers of cultures positive/numbers tested.

### Expression of non-variable VlsE by *B*. *burgdorferi* results in an impaired ability to colonize *Ixodes scapularis*

**Fig 1:**
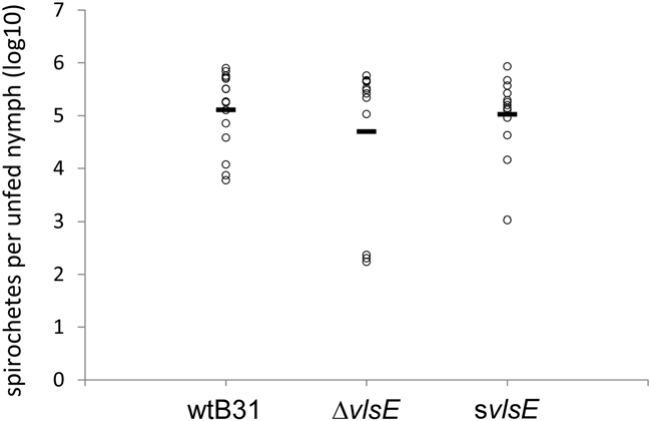
Total spirochete loads of *vls* mutant *B*. *burgdorferi*-infected *Ixodes scapularis*. Spirochetes in unfed nymphs were quantified by qPCR using a primer and internal probe for *flaB*. The number of spirochetes (log10) for each individual unfed tick is shown as an open circle. The black horizontal bar given for each *B*. *burgdorferi* group represents the overall mean. Only PCR-positive samples are included in this Fig. No statistical difference was observed between wtB31 (n = 15) and Δ*vlsE* (n = 12) or wtB31 (n = 15) and s*vlsE* (n = 13) in unfed nymphs, as determined by a two tailed t-test.

In order to examine if the *vls* mutant clones have the capacity to be acquired by *I*. *scapularis* ticks during feeding, naïve C3H/HeNHsd (C3H) mice were subcutaneously inoculated with either wild-type or *vls* mutant *B*. *burgdorferi* at 1.1x10^4^ total spirochetes per animal. All mice became infected with each respective *B*. *burgdorferi* clone as determined by culture-positive blood samples taken at day 6 post infection (data not shown). Approximately 200 *I*. *scapularis* larvae were applied onto each infected mouse at day 6 post infection. Forty-eight hours later, replete larvae were collected during a two-day period, and samplings were individually crushed and cultured in BSK media in order to determine the acquisition rate of fed larvae. Culture of individual replete ticks showed that there was no statistical difference in the infectivity rates among the tested *B*. *burgdorferi* clones, with the single exception of the s*vlsE* mutant expressing a static form of VlsE (Table 4). The acquisition rate of the s*vlsE* clone was found to be 75% compared to 93% and 97.5% for wtB31 and Δ*vlsE*, respectively (p = 0.04). The remaining infected larvae were allowed to molt into nymphs. Randomly-selected, individual nymphs from each group were then crushed and cultured in BSK media in order to determine the capacity of the *B*. *burgdorferi* clones to survive through the tick molting period. In general, the *B*. *burgdorferi* transstadial survival rates correlated well with the acquisition rates, suggesting that both the wild-type and *vls* mutant clones were equally capable of persisting in the tick during the molting period (Table 4). At the same time, there was no difference between spirochete loads of unfed nymphs infected either with Δ*vlsE* or s*vlsE* and those of wtB31-infected nymphs as determined by quantitative PCR analysis (Fig 1), indicating that the *vls* system is unlikely to be involved in *B*. *burgdorferi* survival during molting. Importantly, the negative control samples (DNA extracted from a pool of 50 uninfected ixodid larvae) remained PCR-negative. Together, the data demonstrate reduced tick infectivity rates for the s*vlsE* clone, suggesting that expression of static VlsE leads to diminished acquisition of *B*. *burgdorferi* from a laboratory strain of mice (C3H) by the arthropod vector.

**Table 4:** Infectivity rates of *B*. *burgdorferi* in *Ixodes scapularis* larvae fed on C3H, *P*. *maniculatus* and SCID mice and respective *B*. *burgdorferi* survival rates in flat nymphs.

*B*. *burgdorferi* clone	C3H		*P*. *maniculatus*		SCID	
	Larvae	Nymphs	Larvae	Nymphs	Larvae	Nymphs
wtB31	28/30^a^ (93)	29/30 (97)	14/20 (70)	12/20 (60)	20/20 (100)	17/20 (85)
Δ*vlsE*	39/40 (97.5)	39/40 (97.5)	8/20 (40)	11/20 (55)	20/20 (100)	20/20 (100)
s*vlsE*	30/40 (75)^b^	25/35 (71)^b^	5/20 (25)^b^	5/20 (25)^b^	19/20 (95)	20/20 (100)
297	39/40 (97.5)	40/40 (100)	NA^c^	NA	NA	NA

^a^ Ticks positive for *B*. *burgdorferi* /total numbers of ticks tested by culture (percentage of positives). Total number of ticks is from two separate feeding experiments.

^b^ Denotes statistical significance at p<0.05.

^c^ NA denotes not assessed.

In order to determine if the s*vlsE* clone would also be impaired in acquisition by ticks feeding on a natural murine reservoir, a similar experiment was performed using *P*. *maniculatis* mice. The animals were needle inoculated subcutaneously with either wtB31, Δ*vlsE* or s*vlsE B*. *burgdorferi* clones at 1.1x10^5^ total spirochetes per animal. At day 6 post infection, blood samples were collected from each mouse, and then mice were exposed to approximately 200 *I*. *scapularis* larvae. All animals were culture-positive for *B*. *burgdorferi* clones via blood samples taken at day 6 post infection (data not shown). Samplings of collected replete larvae and molted nymphs were individually crushed and cultured in BSK media. Overall, the acquisition and transstadial survival rates of wtB31, Δ*vlsE* and s*vlsE B*. *burgdorferi* clones obtained from the *Peromyscus* model were found to be sharply reduced compared to those previously observed in C3H mice (Table 4). The *vls* mutants showed a reduction in both rates by approximately 42–50%, whereas acquisition and transstadial survival rates of wild-type *B*. *burgdorferi* were reduced by 23 and 37%, respectively. Interestingly, the acquisition and transstadial survival rates of the s*vlsE* mutant clone were significantly lower than that of wtB31 (p<0.02), suggesting that the presence of the intact *vls* system was advantageous for *B*. *burgdorferi* transmissibility from the mammalian reservoir host to the arthropod vector during an early phase of infection. Although the acquisition rate was also noticeably lower for the Δ*vlsE* mutant clone compared to the wild type, it was not found to be statistically significant (p = 0.06) and there was no significant difference between the rates of Δ*vlsE* and *svlsE* (p = 0.32).

The impaired acquisition of the s*vlsE B*. *burgdorferi* mutant exhibited in the two murine models, C3H and *P*. *maniculatus*, could be accounted for by the host immune response. In order to determine if an antibody-mediated response was responsible for the reduced infectivity rate exhibited by the s*vlsE* mutant clone, C.B-17/IcrHsd-*Prkdc* ^*scid*^ (SCID) mice that lack antibody production were used to assess tick acquisition. SCID mice were subcutaneously inoculated with either wtB31, Δ*vlsE* or s*vlsE* clones, and then exposed to *I*. *scapularis* larvae as described above. The data showed that the acquisition and transstadial survival rates of wtB31, Δ*vlsE* and s*vlsE* by *I*. *scapularis* ticks all fell within an 85–100% range (Table 4), suggesting that VlsE variability is dispensable for efficient tick acquisition from mice when a host antibody response is absent.

**Fig 2:**
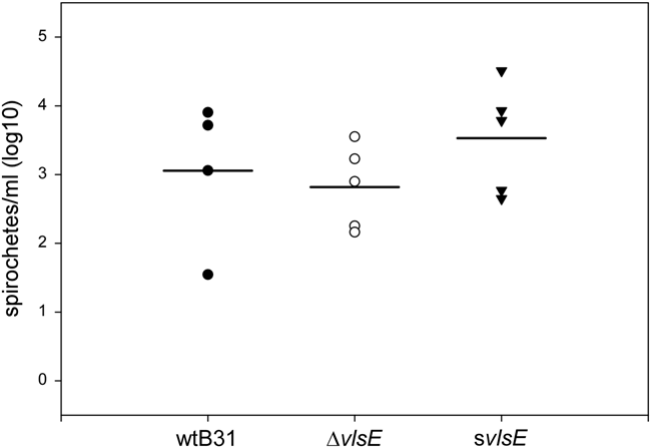
*vls* mutant clones do not show reduced spirochetemia in C3H mice. Blood was collected from saphenous and submandibular veins of C3H mice at day 7 post-infection after subcutaneous needle inoculation with *B*. *burgdorferi* clones. Isolated blood was plated in semi-solid BSK by limiting dilution, and colony-forming units (CFU) were enumerated for each strain. CFU/ml blood is shown for each mouse infected with wtB31 (closed circles), Δ*vlsE* (open circles), and s*vlsE* (closed triangles), as well as mean CFU/ml for each group (horizontal lines).

The observed impairment in tick acquisition of the s*vlsE* mutant clone could be due to low spirochetemia during infection of an immunocompetent host. In order to determine whether the bacterial burden of the s*vlsE* clone was decreased during acute spirochetemia, C3H mice were infected by needle inoculation with 1.1x10^4^ spirochetes of either wtB31, Δ*vlsE*, or s*vlsE* clones. Blood from infected animals was isolated at day 7 post infection, plated in semi-solid BSK culture medium, and spirochete burden was determined by counting *B*. *burgdorferi* colony-forming units. Although variation between animals impeded a statistically rigorous analysis, the overall trend did not show any evidence of decreased spirochetemia for the s*vlsE* clone (Fig 2). In fact, the absolute mean number of spirochetes per ml for the s*vlsE* clone was higher than that of either the wild-type or Δ*vlsE* clones. These data indicate that the impaired tick acquisition of the s*vlsE* mutant clone was likely not due to decreased spirochetemia in the mouse host.

Previous studies have implicated several other plasmid-encoded genes in being important for infection of the tick vector during the enzootic cycle [28,38,39]. Therefore, differences in tick acquisition observed between clones could be due to differential plasmid loss during mouse infection. For this reason, PCR analysis of the plasmid profiles of mouse-passaged colonies isolated from semi-solid BSK was conducted. Interestingly, the results showed that roughly 50% of both the Δ*vlsE* and s*vlsE* clones recovered from the infected mice had lost the truncated or *in cis* complemented lp28-1 plasmid (data not shown). Previous studies have demonstrated that *I*. *scapularis* ticks can acquire and be infected by *B*. *burgdorferi* clones lacking lp28-1 at levels similar to wild-type spirochetes [27,28]. Given that a statistically significant decrease in acquisition by ticks was seen with only the s*vlsE* clone, along with an equal tendency of both *vls* mutants to lose their modified lp28-1 plasmids, these results further indicate that an intact *vls* locus is advantageous for efficient tick acquisition of *B*. *burgdorferi* from the mammalian reservoir. Moreover, this also suggests that the observed impairment in tick acquisition of the s*vlsE* clone may actually underestimate the full detrimental effect of static VlsE expression by *B*. *burgdorferi* for efficient tick acquisition.

### The *vls* system is not sufficient for ‘enzootic’ host reinfection by homologous *B*. *burgdorferi* clones

It was recently reported that an intact *vls* locus is absolutely required for *B*. *burgdorferi* reinfection in laboratory mice using host-adapted spirochetes [15]. Based on the results from this study, it was proposed that VlsE is specifically involved in the evasion of non-VlsE surface antigens from the acquired humoral immune response. As opposed to host-adapted clones, it has been shown that very few spirochetes (<1%) express VlsE in ticks [40], and *vlsE* recombination does not occur during infection of the tick vector [41]. Thus, the expectation would be that tick-transmitted wtB31 would be unable to reinfect mice that had naturally cleared infection with the homologous B31-Δ*vlsE* clone due to absence of the putative protective effects provided by VlsE. In order to test this, 15 naive *P*. *maniculatis* mice were first needle inoculated with the Δ*vlsE* clone. Initial infection was confirmed in all animals either by positive cultures of blood or Western blot analysis of sera sampled at day 7 or 28, respectively. All animals cleared Δ*vlsE* as determined by culture-negative ear biopsies sampled at day 21 and 28 post infection (data not shown). The Δ*vlsE*-exposed mice were then divided into 4 groups, and challenged with either nymph-transmitted wtB31 (5 mice), 297 (4 mice), Δ*vlsE* (3 mice) or s*vlsE* (3 mice). To assess the outcome of reinfection, blood and ear biopsies taken at days 7 and 21 post reinfection were cultured in BSK media. At day 28 post reinfection, tissues (ear, heart, bladder, and tibiotarsal joint) were harvested for culture. The results showed that none of *B*. *burgdorferi* B31 clones had the capacity to reinfect *P*. *maniculatus* mice (Table 5). However, the heterologous wild-type 297 *B*. *burgdorferi* strain was able to reinfect and establish a persistent infection in all 4 out of 4 mice initially infected with the B31 Δ*vlsE* clone. Therefore, as predicted, these results suggest that tick-transmitted wild-type *B*. *burgdorferi* are unable to establish intrastrain reinfection in *Peromyscus* mice. Moreover, the findings reported here provide evidence that reinfection of the host mouse reservoir in nature likely occurs only with spirochetes that are heterologous to the original infecting strain.

**Table 5:** Assessment of reinfection by tick-transmitted *B*. *burgdorferi* in *P*. *maniculatus* mice that previously cleared *in vitro*-grown Δ*vlsE* spirochetes.

Tissue collected (at day post challenge)	Mice that had cleared Δ*vlsE* and reinfected with tick-transmitted:			
	wtB31	Δ*vlsE*	s*vlsE*	297
Blood (day 7)	0/5 ^a^	0/3	0/3	4/4
Ear (day 21)	0/5	0/3	0/3	4/4
Ear (day 28)	0/5	0/3	0/3	4/4
Heart (day 28)	0/5	0/3	0/3	4/4
Bladder (day 28)	0/5	0/3	0/3	2/4
Joint (day 28)	0/5	0/3	0/3	3/4

^a^ Values listed correspond to numbers of cultures positive/numbers tested.

### VlsE-mediated resistance to host antibodies depends on the *B*. *burgdorferi* adaptation state

**Fig 3:**
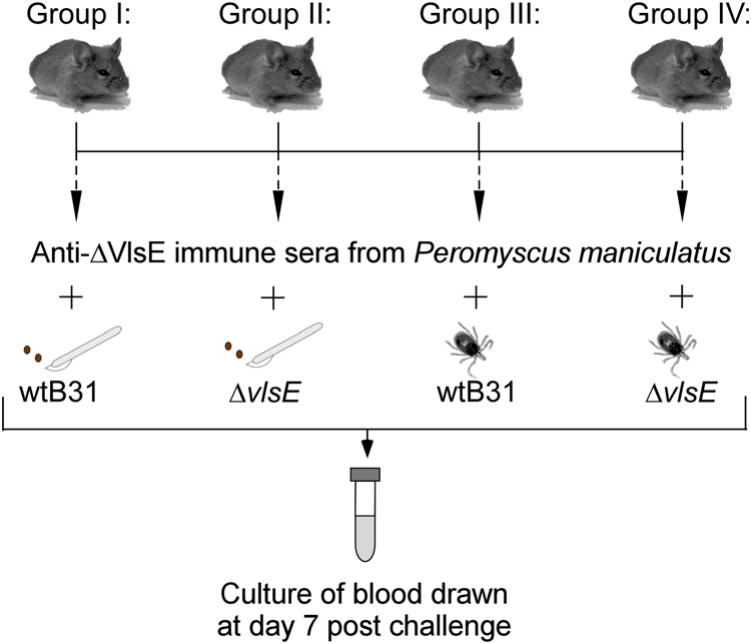
Design of passive transfer experiment to assess the ability of *B*. *burgdorferi* clones to evade antibodies. Preimmune and immune sera were originated, respectively, from naïve and Δ*vlsE*-infected *P*. *maniculatus* mice. Four groups of SCID mice (3 animals per group) were treated with immune sera from Δ*vlsE*-infected mice and challenged 18 hours later with host-adapted wtB31 (group I), host-adapted Δ*vlsE* (group II), nymph-transmitted wtB31 (group III) or nymph-transmitted Δ*vlsE* (group IV) clones. At day 7 post infection, blood was drawn from each mouse and cultured to assess the outcome of challenge. The control groups of mice confirmed the ability of host-adapted Δ*vlsE* or nymph-transmitted wtB31 and Δ*vlsE* clones to infect preimmune sera-treated or naïve SCID mice, respectively.

A state of spirochete adaptation at the time of secondary challenge (i.e. host- vs. tick-adapted *B*. *burgdorferi*) and/or utilization of different murine models (C3H vs. *P*. *maniculatus*) could potentially account for the inability of tick-transmitted homologous *B*. *burgdorferi* to reinfect Δ*vlsE*-exposed *P*. *maniculatus* mice. Recently, it was shown that SCID mice treated with immune sera from Δ*vlsE*-infected mice were resistant to infection by the host-adapted B31-Δ*vlsE* clone, but could be successfully challenged by host-adapted wtB31 spirochetes [15]. Again, because VlsE expression levels are known to be low in *B*. *burgdorferi*-infected ticks relative to that during host infection [36,40–42], the expectation is that tick-transmitted wtB31 would be unable to challenge SCID mice treated with immune sera from Δ*vlsE*-infected mice. To test this, a passive transfer experiment was designed that involved 7 groups of SCID mice (3 animals per group) treated with sera obtained from *P*. *maniculatis* mice (Fig 3). Twelve SCID mice were treated with immune sera from Δ*vlsE*-infected mice, while 9 SCID mice were administered preimmune sera from naïve mice. At 18 hours post treatment, SCID mice were challenged with either host-adapted wtB31 or Δ*vlsE* clones. At day 7 post challenge, blood was drawn from each mouse and cultured to assess the outcome of challenge. The results showed that culture-positive spirochetemia was detected in 3 out of 3 mice challenged with host-adapted wtB31 (group I; Table 6), which was in agreement with previously published findings involving laboratory strains of mice [15,43]. In contrast, passively immunized mice were all resistant to challenge by host-adapted Δ*vlsE* spirochetes (group II; p<0.05). Control SCID mice that received preimmune sera treatment were successfully infected by Δ*vlsE* mutant *B*. *burgdorferi* (group V: p<0.05), indicating that the Δ*vlsE* clone was infectious.

**Table 6:** Infectivity of host-adapted or tick-transmitted *B*. *burgdorferi* clones in SCID mice passively immunized with immune sera from Δ*vlsE*-infected mice.

Group #	Mice treated with sera derived from *P*. *maniculatus* or untreated mice	Challenged with:	Blood at (day 7)	Tissues^a^ (day 28)
I	SCIDs + Δ*vlsE*-specific sera	ha^b^ wtB31	3/3^c^	12/12
II	SCIDs + Δ*vlsE*-specific sera	ha Δ*vlsE*	0/3	0/12
III	SCIDs + Δ*vlsE*-specific sera	tick-transmitted wtB31	0/3	0/12
IV	SCIDs + Δ*vlsE*-specific sera	tick-transmitted Δ*vlsE*	0/3	0/12
V	SCIDs + preimmune sera (control)	ha Δ*vlsE*	3/3	12/12
VI	untreated *P*. *maniculatus* (control)	tick-transmitted wtB31	3/3	NA ^d^
VII	untreated *P*. *maniculatus* (control)	tick-transmitted Δ*vlsE*	3/3	NA

^a^ Tissues harvested and cultured from each mouse include ear, heart, bladder and tibiotarsal joint.

^b^ ha denotes host-adapted clone.

^c^ Values listed correspond to numbers of cultures positive/numbers tested.

^d^ NA denotes not assessed.

To assess the ability of tick-transmitted wtB31 to challenge immune sera-treated mice, SCID mice treated with *P*. *maniculatis* sera were challenged with ticks infected with either wtB31 or Δ*vlsE* spirochetes. The results showed that all immune-sera treated mice were resistant to any challenge by tick-transmitted wtB31 or Δ*vlsE* clones (groups III and IV, respectively; Table 6). Ear, heart, bladder, and tibiotarsal joint tissues harvested from non-spirochetemic animals at day 28 post challenge remained culture-negative for *B*. *burgdorferi*, suggesting that even wild-type *B*. *burgdorferi* were incapable of challenging passively-immunized mice when infecting from a tick-derived state. Both nymph-transmitted wtB31 (group VI) and Δ*vlsE* (group VII) were capable of infecting preimmune sera-treated SCID mice (p<0.05), as shown by positive blood cultures sampled at day 7 post infection (Table 6). Together, the above data indicate that the adaptation state of infecting spirochetes, perhaps due in part to its respective effects on VlsE expression, can greatly influence *B*. *burgdorferi* avoidance from the host antibody-mediated response. Thus, these findings provide further support of previous data [13,15,44] that indicated a VlsE-mediated immune avoidance system may be at work in *B*. *burgdorferi*.

## Discussion

### Importance of the *vls* locus for *B*. *burgdorferi* infection of the mammalian reservoir host

**Fig 4:**
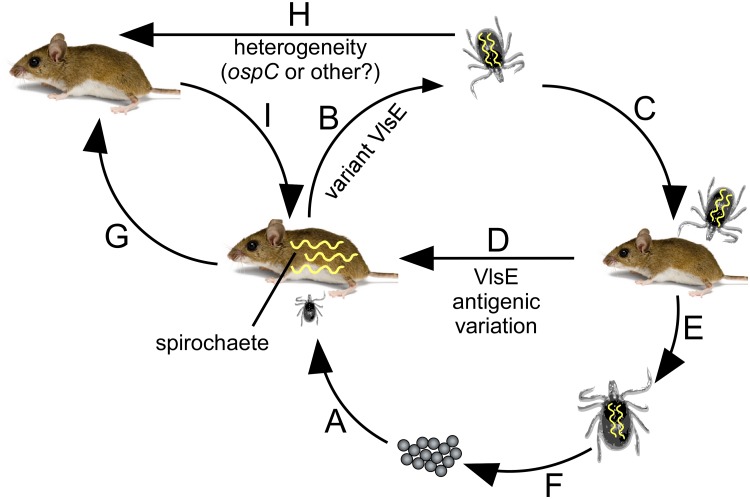
Summary of the importance of VlsE antigenic variation during the enzootic cycle of *Borrelia burgdorferi*. The stages (listed as A through I) of the life cycle of *B*. *burgdorferi* involving the tick vector and reservoir host are shown. A) Acquisition of spirochetes occurs when hatched tick larvae feed on infected *Peromyscus* mice during the summer months. B) The production of VlsE variants by spirochetes is necessary to escape anti-*Borrelia* antibodies present in the bloodmeal, allowing for efficient acquisition by larval ticks that are transstadially retained when the larvae molt into nymphs. C) Infected nymphal ticks transmit spirochetes while feeding on young, uninfected mice during the spring. D) Antigenic variation of VlsE by infecting *B*. *burgdorferi* in these young mice allows spirochetes to persist at least until the summer months in order to be acquired by tick larvae, thereby perpetuating the life cycle of the pathogen. E and F) Infected nymphs molt into adult ticks, which are not considered to be important for maintaining *B*. *burgdorferi* in nature. Adults typically feed and mate on large mammals such as deer, resulting in the next generation of tick vectors. G, H and I) Although not well studied, it is possible that immune clearance of spirochete infection occurs in certain numbers of mice. These mice may become reinfected by feeding nymphs that carry a strain of *B*. *burgdorferi* that is heterogeneous in some way to the original infecting strain. This capacity for reinfection might be highly advantageous for maintenance of the pathogen during ecological situations when immunologically naïve mammalian reservoir populations are of limited availability.

Animal models have been used extensively in order to gain insight on numerous aspects of Lyme disease [45–49]. The findings on the importance of the *vls* locus for host infection thus far have been mainly obtained from various laboratory mouse strains [22–25]. Moreover, the relevance of VlsE antigenic variation for *B*. *burgdorferi* persistence in nature during the enzootic life cycle of the pathogen had not yet been established. Thus, the present study examined involvement of the *vls* locus for the ability of *B*. *burgdorferi* to be acquired by the tick vector, and to be transmitted to and persist in the natural murine host. The findings reported here (summarized in Fig 4) show that devoid of the *vls* locus, both *in vitro*-grown and tick-transmitted *B*. *burgdorferi* lost the capacity to persist in *Peromyscus* mice. This is consistent with previous studies that showed an inability of *in vitro*-grown or host-adapted Δ*vlsE* mutant *B*. *burgdorferi* to establish a persistent infection in laboratory strains of mice [13,15]. The current data also demonstrated that the presence of the *vlsE* expression site alone (without the silent cassettes) did not ensure persistence in the natural host, reiterating the significance of VlsE antigenic variability for sustained infection by *B*. *burgdorferi* [13–15].

The inability of the VlsE-deficient clone to persist in the murine host has been attributed to a failure by the Lyme pathogen to escape the humoral immune response [15,24,25]. The *vls* system has been shown to allow host-adapted spirochetes to specifically evade *B*. *burgdorferi*-specific host antibodies during murine reinfection [32]. Consistently, the current data demonstrated that antibodies generated in the natural reservoir were also borreliacidal to *B*. *burgdorferi* in the absence of the *vls* locus. Infection of SCID mice with the host-adapted VlsE-deficient clone was prevented by Δ*vlsE*-specific antibodies derived from *Peromyscus* mice, whereas host adapted wild-type *B*. *burgdorferi* was able to establish infection in the immunized animals. In contrast, an intact *vls* system was not sufficient for tick-transmitted wild-type *B*. *burgdorferi* to resist Δ*vlsE*-specific immunoglobulins, indicating that VlsE is unlikely to be functionally involved at the time of tick-mediated *B*. *burgdorferi* transmission. Indeed, *vlsE* recombination does not occur during infection of the tick vector [41], and very few spirochetes (<1%) express VlsE [40]. Moreover, the level of VlsE expression is low in ixodid nymphs compared to that found during murine infection, which further supports the insignificant role of the *vls* locus during the initial tick and murine host interaction [36,40–42]. Overall, the present findings agree with the previous study that utilized Δ*vlsE*-specific antibodies originated from C3H mice [15], and provide further evidence in favor of a VlsE-mediated immune avoidance system that has been proposed to prevent *B*. *burgdorferi* surface antigens from being recognized by host antibodies once host infection has been established [13,15,44,50,51].

### The impact of VlsE variability on *B*. *burgdorferi* infectivity of the arthropod host

The *B*. *burgdorferi* enzootic cycle depends on efficient infection of not only the vertebrate host, but also of the arthropod vector in order to ensure continual maintenance of Lyme spirochetes in nature. The findings presented herein demonstrated that *I*. *scapularis* larvae were able to acquire all *B*. *burgdorferi* clones tested, including the *vls* mutants, Δ*vlsE* and s*vlsE*. However, the acquisition rate for the s*vlsE* mutant was found to be significantly lower when larvae were allowed to feed on either C3H or *P*. *maniculatus* mice, demonstrating that the presence of non-switchable *vlsE* impaired the ability of these mutant spirochetes to infect larval ticks. In contrast, the VlsE-deficient mutant was able to be acquired by tick larvae from infected C3H mice at levels comparable to the wild type. This latter finding correlates well with previously published data that demonstrated unimpaired tick acquisition of lp28-1-deficient *B*. *burgdorferi* clones [27,28]. The higher tick acquisition rates of Δ*vlsE*, as opposed to those of s*vlsE*, from immunocompetent mice potentially indicate that a static VlsE variant constitutes a specific target of host antibodies present in the murine blood meal. This is supported by results showing that the s*vlsE* clone exhibits an acquisition rate comparable to that of wild-type *B*. *burgdorferi* when ticks were fed on SCID mice lacking an effective antibody response. The transstadial survival rates of both wild-type and VlsE mutant *B*. *burgdorferi* clones in flat nymphs correlated well overall with the corresponding acquisition rates. This not only served to validate the acquisition rates, but also suggested that the *vls* locus was not required for ticks to remain infected during molting.

Taking into account that acquisition was allowed to occur before the actual onset of an adaptive humoral response [52], it is unlikely that T-cell dependent immunoglobulins were primarily involved in reducing infectivity of tick larvae by the s*vlsE* mutant clone. Natural immunoglobulins that are independent of CD4+ T cells and continuously produced in the murine host [53] were previously shown to be protective in naïve mice against *B*. *burgdorferi* challenge [54]. Moreover, VlsE has been proposed to act as a T-cell independent (TI) antigen due to its immunodominant nature, and may act to directly stimulate B1 subsets [13,50]. Recent data has also shown that immune sera generated from wild type *B*. *burgdorferi*-infected Hsd:Athymic Nude-*Foxn1* ^*nu*^ mice that produce only natural immunoglobulins [55] was able to prevent challenge by clones expressing non-variant VlsE, but could not provide protection against spirochetes that expressed either variable VlsE or no VlsE at all [15]. Thus, it is plausible that either a complete absence or fully functional variant-generating capacity of the *vls* system is required for *B*. *burgdorferi* to evade TI antibodies and, therefore, efficiently infect the arthropod host.

The results from the tick acquisition studies reported here also found that infectivity rates of all *B*. *burgdorferi* clones tested were sharply diminished for ticks fed on *Peromyscus* mice as opposed to those obtained from C3H or SCID mice. Specifically, the acquisition and transstadial survival rates of wtB31 were reduced by 23% and 37%, respectively, whereas the *vls* mutants exhibited even a more pronounced decrease (42.5% to 50%). The impaired ability to infect ticks by all the tested *B*. *burgdorferi* clones that were specifically derived from *P*. *maniculatus* may indicate that spirochete titers are generally lower in the natural reservoir host than laboratory strains of mice. Quantification of the bacterial burden for each *B*. *burgdorferi* clone in murine blood of C3H mice demonstrated that there was no significant difference between the absolute mean numbers of spirochetes per ml for either of the *vls* mutant clones compared to the wild type. These data indicate that the impaired tick acquisition of the static VlsE-expressing clone cannot likely be attributed to decreased spirochetemia in the mouse host. Finally, PCR analysis of the plasmid profiles of mouse-passaged colonies isolated from semi-solid BSK revealed that 50% of either *vls* mutant clone had lost their respective genetically-modified lp28-1 plasmid. This could suggest that the results obtained in the present study actually represent an underestimate of the overall negative effect of static VlsE expression on tick acquisition. However, it is also possible that *in vitro* propagation of a very small subpopulation of *vls* mutant spirochetes that had spontaneously lost the modified lp28-1 plasmid occurred during the initial 12-hour incubation, plating of blood samples on semi-solid medium, and/or subsequent growth of isolated colonies in culture prior to PCR analysis. Nevertheless, the significant reduction in tick acquisition exhibited by the s*vlsE* mutant clone suggests that, in the long run, the presence of a fully functional *vls* system is an obligate requirement for Lyme spirochetes to be successfully propagated through continuous *B*. *burgdorferi* enzootic cycles.

### Secondary challenge by the Lyme pathogen during the *B*. *burgdorferi* enzootic cycle

Secondary infections by the Lyme pathogen are common in nature. Human cases of reinfection are regularly reported in post-treatment patients, indicating that individuals that have successfully recovered from early Lyme disease remain at risk for reinfection [56–64]. Moreover, mixed infections as a result of co-infection or superinfection with various *B*. *burgdorferi* genotypes have been reported in questing ticks [65–67], reservoir animals [68] and humans [69]. The capacity to establish secondary infection might be highly advantageous for *B*. *burgdorferi* to be maintained in the enzootic cycle during ecological situations when immunologically naïve mammalian reservoir populations are of limited availability. Additionally, a recent study used reinfection of mice to determine whether VlsE is specifically involved in evasion of non-VlsE surface antigens from the acquired humoral immune response [15]. Thus, a goal of the present work was to further test this hypothesis using the *B*. *burgdorferi* enzootic cycle model.

Due to the fact that the level of VlsE expression is low in *I*. *scapularis* nymphs [40], the expectation was that tick-transmitted wild-type *B*. *burgdorferi* would be unable to reinfect mice initially infected with a homologous clone. Indeed, the data from the reinfection assays presented herein showed that tick-transmitted wtB31 spirochetes were unable to reinfect B31Δ*vlsE*-exposed animals, which provides further support for the hypothesis that VlsE is required for immune avoidance of non-VlsE surface antigens from the host immune response. Interestingly, when a heterologous 297 strain was used in the reinfection assay, all mice were able to establish culture-detectable spirochetemia followed by a persistent infection. The ability of tick-transmitted heterologous *B*. *burgdorferi* to reinfect mice might indicate that, in the absence of high levels of VlsE, expression of outer surface protein C (OspC) is required to establish reinfection. OspC is an outer surface protein that is upregulated in the feeding tick, and is critical for tick-transmitted *B*. *burgdorferi* to establish infection in the mammalian host [70–75]. Heterogeneity is well-known to exist between the *ospC* genes of different *B*. *burgdorferi* strains [67,76–79], and this could explain why antibodies to B31-specific OspC generated during initial infection were not protective against 297 challenge. However, it has also been reported that homologous *B*. *burgdorferi* clones were capable of reinfecting mice when incubated with the tick salivary protein, Salp15, which is known to bind to OspC [80]. This may suggest that OspC is not the critical factor in determining host reinfection, and that some other factor(s) are responsible.

In summary, the present study provides the first direct evidence for the significance of VlsE during the *B*. *burgdorferi* enzootic cycle. This work is also the first to examine the ability of tick-transmitted *B*. *burgdorferi* to reinfect a known competent murine host in nature. The current data demonstrates an absolute requirement of the *vls* locus for *B*. *burgdorferi* to establish a persistent infection in the *B*. *burgdorferi* reservoir host, *Peromyscus maniculatis*. Together, the findings of this study suggest that the variant-generating capacity of the *vls* system is crucial for the Lyme pathogen to be efficiently and successfully perpetuated throughout the *B*. *burgdorferi* life cycle.
